# United Nations Partnerships With the Alcohol Industry

**DOI:** 10.34172/ijhpm.8947

**Published:** 2026-01-28

**Authors:** June Yue Yan Leung, Sally Casswell

**Affiliations:** SHORE and Whariki Research Centre, College of Health, Massey University, Auckland, New Zealand.

**Keywords:** Alcohol, Corporate Social Responsibility, Industry, Partnership, United Nations

## Abstract

**Background:**

The alcohol industry builds engagement with United Nations (UN) organisations to enhance its corporate image and influence policy, supported by the UN’s endorsement of public-private partnerships (PPPs). However, the extent of the alcohol industry’s relationships with the UN remains unclear due to limited reporting.

**Methods:**

We searched the websites of 57 UN-affiliated entities and 18 transnational alcohol corporations (TNACs) for evidence of partnerships or relationships between the UN and the alcohol industry. We summarised the UN entities and TNACs involved in formal partnerships, membership of alliances or stakeholder networks, financial contributions, sponsorship of programmes or projects, sponsorship of events, event participation, and personal relationships with conflicts of interest.

**Results:**

We identified examples of all the above relationships between various UN entities and the world’s largest TNACs, including an alcohol industry donation towards the World Health Organization (WHO) Foundation, which was created to maximise private sector donations to WHO. The focus of these engagements aligned closely with the alcohol industry’s corporate social responsibility (CSR) initiatives, including drink-driving prevention, education, sustainability, and philanthropy. These activities frequently involved support for low- and middle-income countries (LMICs) and women, which are emerging markets for the TNACs. Sponsorship and participation in intergovernmental events allowed the TNACs privileged access to policy-makers. Limited disclosure by UN entities meant that our findings provided an incomplete picture of relationships with the alcohol industry.

**Conclusion:**

The UN’s wide-ranging relationships with the TNACs highlight the power of these large corporations in building political influence and the UN’s failure to acknowledge the alcohol industry’s conflicting interests with health. These relationships undermine WHO’s mandate to promote health, placing the integrity and impartiality of the UN system at risk. On top of adequate resources from member states and enhanced transparency measures, the UN requires effective safeguards against alcohol industry influence, in line with those for the tobacco industry.

## Background

**Key Messages**

**Implications for policy makers**

- The alcohol industry builds engagement with the United Nations (UN) in order to promote its corporate image and influence policy, supported by the UN’s endorsement of public-private partnerships (PPPs).
- We found a wide range of partnerships and relationships between various UN entities and the world’s largest alcohol corporations, including a donation from the alcohol industry to support the World Health Organization (WHO).
- As alcohol is a leading cause of harm and the alcohol industry derives much of its profits from heavy drinking, these relationships undermine WHO’s mandate to promote health and the UN’s commitment to reduce the burden of non-communicable diseases (NCDs).
- To protect the integrity and impartiality of the UN, governments should provide the UN with the resources and safeguards necessary against alcohol industry influence, in line with those for the tobacco industry.

**Implications for the public**

The alcohol industry uses sophisticated strategies to build its corporate reputation and influence policy to serve its interests. A key strategy is to develop long-term relationships with the United Nations (UN), providing the industry with access to policy-makers. Our study identified a wide range of relationships between various UN entities and the world’s largest alcohol corporations, including partnerships, membership of alliances or stakeholder networks, financial contributions, sponsorship of activities or events, event participation, and personal relationships with potential conflicts of interest. Notably the alcohol industry donated to the World Health Organization (WHO) Foundation, undermining WHO’s mandate to keep the world safe from alcohol harm. To protect the UN’s independence, governments need to provide the UN with adequate resources to do its work. The UN should exclude the alcohol industry from these partnerships and fully disclose its interactions with private companies.

Alcohol industry interference remains common within countries and is a key barrier to the adoption of effective government policies that reduce alcohol harm.^1,2^ As a result of consolidation nationally and internationally, highly profitable transnational corporations now dominate the global alcohol industry, with vast resources to buy into new markets, control distribution, and influence policy.^3^ These companies use corporate social responsibility (CSR) initiatives to enhance their reputation, market their brands and divert attention away from effective policy interventions.^4^ By positioning themselves as “partners” in reducing alcohol harm, such as sponsoring intergovernmental events and education campaigns, the alcohol industry attempts to influence policy by gaining access to policy-makers.^5,6^ These practices are similar to those used by other unhealthy commodity industries, such as tobacco, ultra-processed food and fossil fuels; empowered by governments, intergovernmental organisations, and a global political and economic system that increasingly enables commercially driven health and social harms.^7^

 One aspect of this alcohol industry project is to build engagement in the global arena with United Nations (UN) organisations through public-private partnerships (PPPs). The value of this engagement to industry was acknowledged in a report by the UN Joint Inspection Unit, which summarised the main motivational factors of businesses to partner with the UN: having access to international policy debates, governments and major stakeholders; being associated with the unique image of the UN; developing new markets and business opportunities; fulfilling their CSR or sustainability goals; aligning with the Sustainable Development Goals (SDGs); and building their own capacity.^8^ These strategies are in keeping with the fiduciary duty of alcohol companies to maximise profits for their stakeholders.^5^ In its reports to shareholders, the alcohol industry has also highlighted the importance of this engagement in relation to industry influence on policy development. For example, Diageo described in its annual report, “proactive, evidence based engagement to build trust and deepen our relationship and reputation with governments, industry and other stakeholders... have successfully mitigated threats and built momentum in our engagement with governments around the world *to shape more balanced regulatory outcomes*” (emphasis added).^9^

 The UN’s enthusiasm for PPPs stems from persistent shortfalls in funding that has failed to keep up with the system’s growing responsibilities and mandates.^10^ The establishment of the UN Foundation by a United States (US) billionaire in 1997 brought the UN closer to private funders.^10^ In 2000, the UN Global Compact (UNGC) was launched as a voluntary CSR initiative encouraging businesses to adopt “universal principles” related to human rights, labour, the environment, and anti-corruption; and to take actions to advance broader societal goals, including the SDGs.^11^ UNGC has become one of the few UN entities predominantly dependent on private funding.^10^ UN funding from the private sector has grown steadily since, accounting for 4% of total revenue in 2019.^10,12^ The World Health Organization (WHO) has also called for “increased flexible and predictable financing” to address ongoing budgetary pressures, with about 10% of its funds coming from philanthropic foundations.^13^ With member states reluctant to increase their contributions to WHO,^14^ the WHO Foundation (WHOF) was created in 2020 to diversify funding sources and “trusted implementing partners” available to WHO.^15^ As a legal entity separate from WHO, WHOF explicitly seeks funding to expand WHO’s donor base, including from corporations and high-net-worth individuals.^16^ This has raised concerns about risks of undue corporate influence, including from the alcohol industry.^17^ Nonetheless, WHO remains the main UN policy holder in response to alcohol harm, although its alcohol portfolio receives very limited funding compared to tobacco control.^18^

 As alcohol remains a leading cause of death and disability and the alcohol industry relies on heavy drinking for much of its profits,^5,19^ UN partnerships with the alcohol industry may conflict with WHO’s mandate to promote health. UNGC guidelines defined partnership as a “voluntary and collaborative agreement or arrangement between one or more parts of the UN system and the business sector,” the overarching principle being that collaboration should serve the purposes and principles of the UN Charter, while maintaining the integrity, impartiality and independence of the UN.^20^ The UN Charter is centred on maintaining international peace and security, not health.^21^ However, in the context of inadequate government funding and constraints on government power, PPPs have become the primary method advocated for non-communicable diseases (NCDs) and SDGs.^22,23^ The UN General Assembly’s first political declaration on NCDs in 2011 promoted engagement with the private sector in “collaborative partnerships to promote health and to reduce NCD risk factors.”^24^ In 2015, promoting PPPs was specified as the only mechanism dedicated to implementing the SDGs.^25^ The third political declaration on NCDs in 2018 further called on member states to “continue exploring voluntary innovative financing mechanisms and partnerships” with the private sector.^26^

 The alcohol industry has not been clearly excluded from such PPPs. WHO’s Framework of Engagement with Non-State Actors (FENSA) was developed in 2016 to protect the agency from undue influence, requiring WHO to exercise “particular caution” when engaging with non-state actors whose policies and activities may be harmful to health.^27^ FENSA only precluded WHO from engagement with the arms and tobacco industries. UNSDG guidelines published in 2020 recommended UN organisations not to pursue partnerships with businesses considered “inherently incompatible with values of the UN, its treaties, or other international standards.”^28^ As in FENSA, the arms and tobacco industries were specifically named, but not the alcohol industry. WHO’s internal guidelines in 2019, however, stated that “interaction with the alcohol industry within a given framework should not lead to or imply ‘partnership,’ ‘collaboration,’ or any other similar type of engagement that could give the impression of a formal joint relationship, the reason being that such engagements would put at risk the integrity, credibility, and independence of WHO’s work.”^29^

 Given the limited reporting of UN partnerships with the private sector, the extent of the alcohol industry’s involvement in UN agencies remains unclear. Since 2010, the Secretary-General’s reports on the UN Office for Partnerships (UNOP) have not published the private actors that had partnerships or alliances with the UN.^10^ Disaggregated information on private sector funding is not available and public disclosure requirements are lacking across the UN.^30^ Using publicly available information online, we identified the UN-affiliated entities that have established partnerships or relationships with transnational alcohol corporations (TNACs) and illustrate the range of collaborations involved.

## Methods

 This was an exploratory study that sought to present the breadth of relationships between the UN and the transnational alcohol industry. We aimed to document notable examples of such relationships and to consider their implications for global actions to minimise alcohol harm. Here we focused on the world’s largest TNACs, which are predominantly producers, wholesalers, distributors and marketers of alcohol.^31^

 We conducted a search of 57 UN and 18 TNAC websites to identify evidence of partnerships or relationships. These searches were carried out from July to September 2024. As the first author is proficient in English and Chinese, search results in these langugages were screened for potential relevance. Relevant results were then read and summarised by the UN entity and TNAC involved, year, country, type of partnership, and details of the partnership. As “partnerships” are defined differently across UN organisations,^28^ we examined a broad range of relationships between the UN and TNACs based on prior reviews of alcohol industry strategies to influence policy.^32,33^ Table 1 describes the types of relationships included in this study and how each may be used by the alcohol industry to build long-term relationships with policy-makers. We excluded instances that did not involve clear relationships or engagement between the parties, such as TNAC claims of commitment to UN principles or SDGs.

**Table 1:** Classification of Relationships Between United Nations Entities and Transnational Alcohol Corporations

**Type of Relationship**	**Definition**	**Alcohol Industry Strategy**
Formal partnership	Collaborative agreement involving public recognition of “partnership” between UN entities and TNACs	Securing access to policy-makers
Membership of alliances or stakeholder networks	UN entities and TNACs being members of common alliances or stakeholder networks	Forming business alliances and securing public support for industry positions
Financial contributions	Financial contributions, such as donations and loans, from TNACs to UN entities, or vice versa	Providing incentives to policy-makers
Sponsorship of programmes or projects	TNAC sponsorship or co-hosting of UN programmes or projects, such as training programmes	Creating or sponsoring information favourable to the industry
Sponsorship of events	TNAC sponsorship or co-hosting of UN events, such as workshops or conferences	Creating or sponsoring information favourable to the industry
Event participation	Attendance of TNAC representatives at UN events or meetings, or vice versa	Promoting evidence favourable to the industry
Personal relationships with conflicts of interest	Personal relationships involving TNAC or UN staff that pose potential conflicts of interest, such as revolving doors between positions within TNACs and UN entities	Seeking regulatory capture and access to policy spaces

Abbreviations: TNACs, transnational alcohol corporations; UN, United Nations.

 Table S1 of Supplementary file 1 summarises the 57 UN entities that were examined. UN-affiliated entities included six funds and programmes, 18 specialised agencies, 11 other entities and bodies, five departments and offices, and 12 related organisations. These entities were identified from the UN website and system chart,^34^ supplemented by the authors’ knowledge. We also included five independent charitable organisations that directly fund the UN, such as the UN Foundation and WHOF. As there are numerous UN-related entities, we only included those with potential relevance to the alcohol industry. We used multiple variations of each TNAC’s name as search terms and the search engine on each UN entity’s website, so any relevant results were derived from these organisations’ published materials.

 We identified the TNACs that were on the Forbes Global 2000 List,^35^ which ranked the world’s top companies by revenue, profits, assets and market value (Supplementary file 1, Table S2). Of the 18 TNACs included, nine have their headquarters in Asia (China, Japan, and Thailand), six in Europe (France, Belgium, Denmark, Netherlands, and United Kingdom), and three in North America (US). Twelve were based in high-income economies and six in middle-income economies. Given the global nature of UN partnerships, our search did not specifically cover local alcohol producers, distributors, marketers or retailers, except for subsidiary companies of the above TNACs. We used “United Nations” as the search term on each TNAC’s website, supplemented by a search on Google to identify other relevant articles.

 Here we have summarised the UN-affiliated entities engaged in each type of partnership. Although a formal thematic analysis was not done, we have included detailed examples and quotes to illustrate the range of relationships identified.

## Results

###  Formal Partnerships

**Figure 1:**
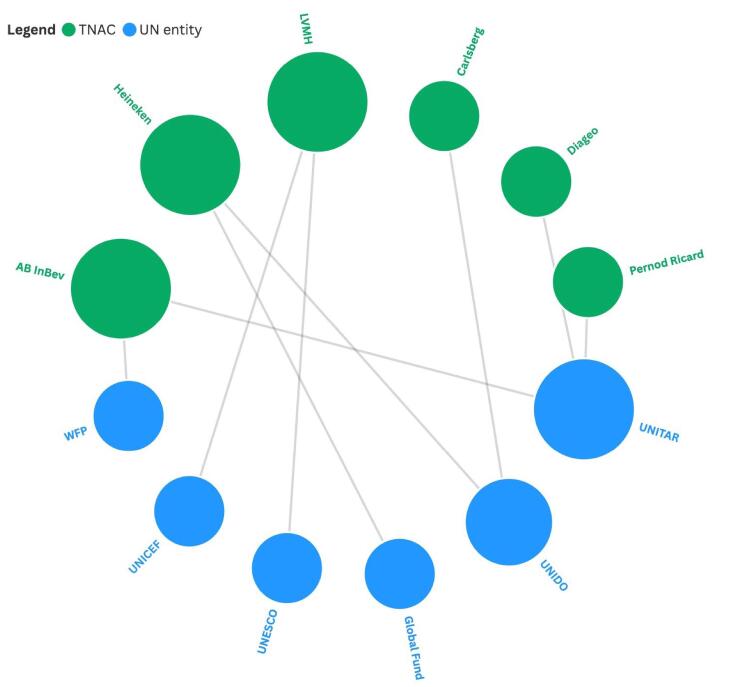
Formal Partnerships Between United Nations Entities and Transnational Alcohol Corporations. Abbreviations: TNAC, transnational alcohol corporation; UN, United Nations; UNESCO, United Nations Educational, Scientific and Cultural Organization; UNICEF, United Nations Children’s Fund; UNIDO, United Nations Industrial Development Organization; UNITAR, United Nations Institute for Training and Research; WFP, World Food Programme.

 We identified six UN entities that have had formal partnerships with six TNACs (Figure 1). These partnerships involved promoting road safety, sustainability or the SDGs, biodiversity, empowerment of women, humanitarian support for children, support for farmers, and communicable disease control.

 The UN Institute for Training and Research (UNITAR) has formal partnership agreements with Anheuser-Busch InBev (AB InBev), Diageo and Pernod Ricard focused on training programmes for road safety and drink-driving prevention.^36-38^ At the signing of the partnership agreement with Diageo in 2016, the UN Assistant Director-General and Executive Director of UNITAR remarked, “Achieving the 2030 Development Agenda and its different goals will only be possible through innovative partnerships with the private sector such as this one, where different stakeholders join forces to reach specific beneficiaries and targets by sharing their respective expertise and resources, thereby achieving common objectives.”^37^ UNITAR renewed its partnership with AB InBev in 2022, adding new initiatives to support female entrepreneurs and sustainable water management practices.^39^ Partnering with UNITAR’s EducateAll platform, Pernod Ricard has also launched a free online “sustainable and responsible bartending training course.”^40^

 The UN Industrial Development Organization (UNIDO) initiated a partnership promoting environmental sustainability with Carlsberg and its Russian subsidiary Baltika Breweries in 2012.^41^ An evaluation by UNIDO described this partnership as helping Carlsberg maintain its “licence to operate,” and that “one of the most important reasons for business partners to collaborate with UNIDO seems to be a wish for reputation gain, visibility, good-will and credibility.”^42^ UNIDO started another partnership with Heineken in 2015, which covered water stewardship, renewable energy and local sourcing initiatives primarily in Africa.^43^ This partnership was described as an “exciting new area of work for UNIDO” and in the words of UNIDO’s Director-General, “Ultimately, we want to improve the lives of people in developing countries and make a meaningful contribution to inclusive and sustainable development while, at the same time, create flourishing markets that foster business opportunities.”^43^ For Heineken, “UNIDO will act as an independent broker, bringing together key stakeholders and helping us develop Public-Private Partnerships where relevant.”^44^ The Global Fund also signed a partnership agreement with Heineken in 2018, with the objective of ending HIV, tuberculosis and malaria as epidemics in Africa.^45^ Heineken was to “lend its expertise in logistics and communications,” using its distribution networks in rural Africa to support the delivery of healthcare supplies.^45^ However, the partnership was suspended shortly afterwards following protest by civil society and specifically attributed to allegations involving Heineken’s use of female beer promoters.^46^

 The UN Educational, Scientific and Cultural Organization (UNESCO) has been in partnership with LVMH Moët Hennessy - Louis Vuitton (LVMH) since 2019, as part of UNESCO’s Man and the Biosphere Programme promoting biodiversity in the Amazon basin.^47^ LVMH is the only private sector partner in this scientific initiative, describing this as an “innovative framework for international cooperation and, beyond the preservation of protected zones, aims to establish best practices for sustainable development to help achieve the UN Sustainable Development Goals.”^48^ Under the same programme, UNESCO and Guerlain, a subsidiary of LVMH, launched a sustainable beekeeping programme for female entrepreneurs.^49^ Louis Vuitton, another subsidiary of LVMH, has also been a corporate partner of UNICEF since 2016.^50^ This partnership mainly involves providing humanitarian support for children.

 The World Food Programme (WFP) has signed agreements with Zambia Breweries and Tanzania Breweries, both subsidiaries of AB InBev, to provide support to local smallholder farmers.^51^ According to a WFP report in 2021, these agreements would serve as case studies for a global WFP-AB InBev partnership that was under negotiation.^51^ In 2024, Accra Brewery, another AB InBev subsidiary, partnered with WFP to support farmers in Ghana, with a focus on empowering women and youth.^52^

###  Membership of Alliances or Stakeholder Networks

**Figure 2:**
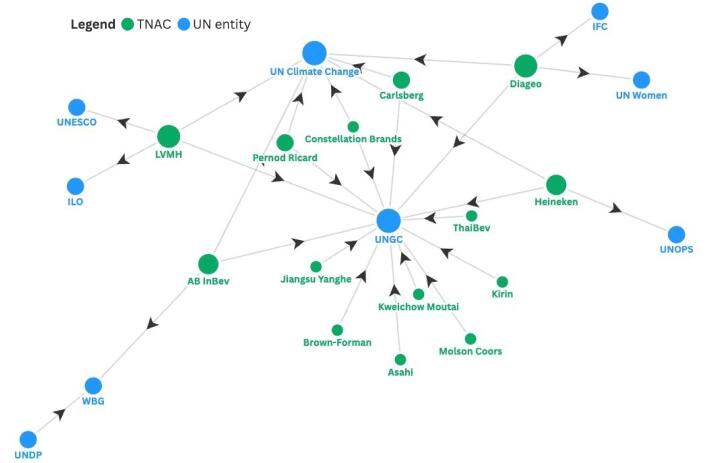
Transnational Alcohol Corporation Membership of United Nations Alliances and Stakeholder Networks. Abbreviations: TNAC, transnational alcohol corporation; UN, United Nations; IFC, International Finance Corporation; ILO, International Labour Organization; UNDP, United Nations Development Programme; UNESCO, United Nations Educational, Scientific and Cultural Organization; UNGC, United Nations Global Compact; UNOPS, United Nations Office for Project Services; WBG, World Bank Group.

 Eight UN entities had alliances or stakeholder networks that 14 TNACs have been members of (Figure 2). These alliances involved themes similar to those above, primarily promoting sustainability and empowerment of women.

 UNGC has had at least 14 TNAC participants, including AB InBev, Asahi, Brown-Forman, Carlsberg, Constellation Brands Mexico, Diageo, Heineken, Jiangsu Yanghe, Kirin, Kweichow Moutai, LVMH, Molson Coors, Pernod Ricard, and ThaiBev.^53^ To join UNGC, companies commit to meeting responsibilities in human rights, labour, environment, and anti-corruption; and make an annual financial commitment to support UNGC.^54^ UNGC has also launched the “Water Resilience Coalition,” which AB InBev and Diageo co-founded and Heineken is a member of.^55^ UN Climate Change also initiated several initiatives of which seven of the above TNACs are members.^56^ These alliances were created to facilitate UN commitments to climate action, such as reducing carbon emissions.^57^ In a similar vein, World Bank Group (WBG) has a PPP known as the “2030 Water Resources Group,” which includes AB InBev and UNDP as members.^58^

 WBG’s International Finance Corporation (IFC) has convened a “Sourcing2Equal Colombia” programme to promote business opportunities for women entrepreneurs, which Diageo joined.^59^ Diageo was also a founding member of UN Women’s “Unstereotype Alliance” to promote gender equality in advertising.^60^ Christian Dior Couture, a subsidiary of LVMH, has joined UNESCO’s “Global Education Coalition” to support female students from low- and middle-income countries (LMICs).^61^ The International Labour Organization (ILO) “Global Business and Disability Network,” which promotes workforce practices inclusive of people with disabilities, also includes LVMH as a member.^62^ Finally, Heineken is a member of the Stop TB Partnership, committed to eliminating TB as a public health problem by 2030 and hosted by the UN Office for Project Services (UNOPS).^63^

###  Financial Contributions

 At least five UN entities have received donations from six TNACs (Table 2). In 2022, WHOF received a donation of USD 100 000 from Duty Free Shoppers (DFS) Group, a subsidiary of LVMH that sells wine and spirits.^64^ This donation was earmarked for the “Go Give One” campaign to support the delivery of COVID-19 vaccines to LMICs. The UN Refugee Agency (UNHCR) received more than USD 2 million combined from Pernod Ricard and Carlsberg in 2022.^65^ Pernod Ricard was named as a donor to UNHCR’s Ukraine emergency response.^66^ AB InBev pledged to donate more than USD 2 million to WBG from 2020 to 2025, in support of the 2030 Water Resources Group.^67,68^ UN Women received donations of USD 30 000 from Diageo in 2020 and 2021.^69,70^

**Table 2:** Summary of Financial Contributions Between United Nations Entities and Transnational Alcohol Corporations

**Year**	**Donor **	**Recipient**	**Contribution Amount**
2022	DFS Group	WHOF (“Go Give One” Campaign)	US$100 000
2022	Pernod Ricard	UNHCR (Ukraine emergency response)	US$1 216 419
2022	Carlsberg Breweries	UNHCR	US$755 059
2020-2022	AB InBev	WBG (2030 Water Resources Group)	US$1 500 000
2023-2025	AB InBev	WBG (2030 Water Resources Group)	US$750 000
2020	Diageo	UN Women	US$30 000
2021	Diageo	UN Women	US$30 000
2005	CFC	Sierra Leone Breweries (subsidiary of Heineken) and Guinness Ghana Breweries (then subsidiary of Diageo) (West African Sorghum Value Chain Development project)	US$1 500 000
2017	IFC	Heineken in Ethiopia (improving quantity and quality of smallholder-based malt barley production)	Unspecified (Total project budget US$999 998)
2017	UNDP	Ghana Ministry of Finance, in partnership with Guinness Ghana Breweries (then subsidiary of Diageo)	US$700 000

Abbreviations: DFS, Duty Free Shoppers; UN, United Nations; WHOF, WHO Foundation; UNHCR, United Nations Refugee Agency; WBG, World Bank Group; CFC, Common Fund for Commodities; IFC, International Finance Corporation; UNDP, United Nations Development Programme.

 We also identified three UN entities that funded two TNACs via PPPs. In 2005, the Common Fund for Commodities (CFC) funded a five-year PPP supporting the sorghum production chain in Sierra Leone and Ghana for local subsidiaries of Heineken and Diageo, respectively.^71^ Heineken’s sorghum project was then awarded a “World Business and Development Award” at the UN’s Millennium Development Goals Summit in 2010.^72^ In 2017, IFC supported Heineken in its malt barley production chain in Ethiopia, in the name of “improving the productivity of smallholder farmers.”^73^ This two-year project was developed in partnership with the Netherlands government.^74^ In the same year, UNDP provided US$700 000 to Ghana’s Ministry of Finance to implement a one-year project “promoting inclusive growth and development” in partnership with Guinness Ghana, then a subsidiary of Diageo.^75^

###  Sponsorship of Programmes or Projects

 Two UN entities have had programmes or projects sponsored by five TNACs. AB InBev sponsored UNITAR’s digital platform for alcohol screening and brief interventions and learning app on road safety.^76,77^ As UNITAR’s other partners, Diageo sponsored UNITAR’s online training programmes on road safety,^78,79^ and Pernod Ricard sponsored training programmes to prevent drink-driving in several middle-income countries, such as Cambodia and Dominican Republic.^80,81^ These three TNACs and Heineken also sponsored UNITAR’s “Water Academy” programme focused on implementing the water-related SDGs.^82^ Separately, AB InBev sponsored the UN Environment Programme “Freshwater Challenge,” which aims to restore rivers and wetlands.^83^ Kirin sponsored a “sustainable tea landscapes project” in China, India, Sri Lanka and Vietnam, overseen by the UN Environment Programme.^84^

###  Sponsorship of Events

 Four UN entities have had events sponsored by or co-hosted with five TNACs. In 2014, AB InBev sponsored the first International Telecommunication Union “symposium on the future networked car” to promote road safety.^85^ UNITAR and AB InBev have co-hosted conferences and workshops on road safety in Barbados, China, and South Africa.^86-88^ UNITAR and Diageo have also organised multiple conferences and workshops on road safety in middle-income countries such as Brazil, India and Senegal.^89-91^ In 2021, UNITAR and AB InBev co-hosted an international dialogue for the public and private sectors to support vaccination for COVID-19, which highlighted AB InBev’s CSR initiatives including producing hand sanitiser, donating personal protective equipment and financing the construction of hospitals.^92^ In 2023, UNESCO and LVMH organised a summit on biodiversity, showcasing their partnership activities and LVMH’s CSR strategies on the environment.^93^ Christian Dior Couture, part of LVMH group, also co-hosted the “UNESCO-Women@Dior Global Conference.”^94^ Jiangsu Yanghe’s spirits were featured at a 2024 Chinese New Year event organised by the UNESCO Chinese delegation at UNESCO Headquarters.^95^ In 2023, ILO organised a career fair in Cambodia to promote job opportunities in hospitality and tourism, sponsored by Pernod Ricard.^96^

###  Participation at Events

 At least 24 UN entities have either invited TNAC representatives to participate in their events or been invited by TNACs to their events (Supplementary file 1, Table S3). Some notable instances are highlighted here. Representatives of Diageo, Heineken, and Pernod Ricard have participated in multiple intergovernmental meetings of the joint Food and Agriculture Organization/WHO Codex Alimentarium as part of their countries’ delegations.^97-99^ A Diageo representative was an invited speaker at the UN summit on Millennium Development Goals in 2010, hosted by UNGC, UNDP, and UNOP.^100^ Kirin was one of two Japanese companies that presented at the third session of the Conference of the Parties to the UN Framework Convention on Climate Change in 1997.^101^ The World Trade Organization has organised multiple business leader meetings and conferences on trade, with representatives of Diageo and Pernod Ricard invited as speakers.^102,103^ Heineken has hosted symposia on its work in Africa, with senior executives from UNAIDS as guest speakers.^104,105^ Heineken has also participated in the Global Fund’s annual board meetings and continued to do so in 2019, even after its partnership with the fund was suspended.^106^

###  Personal Relationships With Conflicts Of Interest

 Finally, we identified personal relationships with potential conflicts of interest involving two UN entities and four TNACs. The current WBG President declared directly holding stocks valued above USD 10 000 in AB InBev, Carlsberg and LVMH in 2023, among many other companies including producers of tobacco and ultra-processed food.^107^ Diageo’s Head of Global Alcohol Policy was a member of UNITAR’s Division for People and Social Inclusion Advisory Board, which is responsible for ensuring that “UN guidelines are respected and well-integrated” in the Division’s activities.^108^

## Discussion

 Our findings illustrate wide-ranging relationships between multiple UN entities and the world’s largest TNACs, suggesting that the UN system lacks effective safeguards against alcohol industry influence. These relationships extended far beyond the UNDP and IFC, the UN’s development and financial agencies that are known to finance alcohol production plants in LMICs as part of a broader strategy to stimulate economic development.^109^ Of particular concern was the alcohol industry’s financial contribution to WHOF, as WHO is the only UN entity with a continued interest in reducing alcohol harm. The relationships we identified are in line with the alcohol industry’s CSR initiatives, which have public relations benefits for the industry while appearing to serve a public health purpose.^4^ Consistent with a systematic review on alcohol industry CSR, drink-driving prevention and education were prominent activities, as was philanthropy related to non-alcohol issues, such as humanitarian aid and communicable disease control.^6^ Alignment with the UN SDGs further satisfies the alcohol industry’s business case for CSR to enhance long-term sustainability, reputation, and ultimately, profitability.^110^ Many of the alcohol industry’s activities involved support for LMICs and women, which are also emerging markets for the TNACs. Finally, by sponsoring and partaking in intergovernmental events, the alcohol industry gains privileged access to policy-makers as a means to influence policy.^32^

 To our knowledge, this is the first study to delineate the extent of UN partnerships with the alcohol industry. While the findings here should be considered exploratory, we conducted a search of publicly available information that can easily be replicated. We also included relevant material in Chinese, providing insight into the growing Chinese TNACs, which have been actively participating in UN-related events in recent years, both within China and abroad.

 Nonetheless, our study also had several methodological limitations. First, our findings are not exhaustive, as the search was only conducted by the first author and would not have identified all relationships between UN entities and TNACs. The UN’s lack of mandatory reporting or repositories of PPPs meant that we had to rely on a manual search of their websites for specific TNAC names to maximise relevance. In particular, this process would not have captured all financial contributions, as very few UN entities publish the amounts received from individual corporate donors.^30^ Information on personal conflicts of interest was also likely incomplete, as self-declarations by UN officials appeared to be rarely published. Second, we have only examined search results in English and Chinese for practical considerations. The inclusion of other languages and the use of translation tools may yield additional relevant findings, although as discussed above, this study was only meant to be exploratory. Third, we did not carry out a formal thematic analysis, as the study’s aim was to describe the range of relevant relationships. We have only highlighted relevant examples and quotes to show how relationships were perceived by the UN entities or TNACs involved. Qualitative analysis of this material may provide further insight into these institutions’ incentives in pursuing partnerships.

 The wide-ranging relationships identified here highlight the power of large transnational corporations within a complex system of global governance that appears to be captured by these commercial interests. The TNACs’ access to these policy spaces also illustrate the potential benefits of these collaborations for the alcohol industry, which seeks to influence policy by building long-term relationships with key actors.^32^ Nonetheless, the vast majority of the alcohol industry’s actions, including those to reduce drink-driving, lack scientific support and may even do harm.^4,111^ Rather than reducing harmful drinking, CSR initiatives are used to influence the framing of alcohol-related issues in keeping with the industry’s interests, with powerful implications for policy-making.^6^ Given the strong business links between the alcohol, tobacco and ultra-processed food industries,^112^ the alcohol industry’s activities identified here are comparable to the multifaceted CSR strategies used by other health-harming industries to enhance their reputations, build political influence, and ultimately stop governments and global organisations from adopting effective policies to minimise harm.^33,113^

 The presence of multiple partnerships with the TNACs also suggests a failure by the UN to recognise the alcohol industry’s conflicting interests with health, given heavier drinking contributes to a large share of industry profits.^114^ Alcohol is said to play an “ambiguous role in economic and social development” as the UN has tended to regard alcohol as any other commodity, without considering its risks to health or costs to society.^115^ Nonetheless, UN entities have acknowledged the potential risks of collaborating with the alcohol industry. For instance, WBG published a “note on alcohol beverages” in 2000, requiring support of alcohol-related projects to be “highly selective” and for projects to “demonstrate strong development impacts which are consistent with public health issues and social policy concerns.”^116^ UNDP’s current “policy on due diligence and partnerships with the private sector” classifies the alcohol industry as a “high-risk sector,” which requires a higher level of decision-making and monitoring within the organisation.^117^ A report by the UN Inter-Agency Task Force on the Prevention and Control of NCDs convened by UNDP also identified the alcohol industry as interfering in policy development.^118^ However, we have shown both organisations to have various relationships with the TNACs.

 Given WHOF funds WHO’s work to protect and promote health, WHOF’s acceptance of a donation from the alcohol industry is deeply concerning. While WHO has stated that it would not collaborate with the alcohol industry, WHOF’s “gift acceptance policy” no longer excludes contributions from the alcohol industry.^17,119^ In early 2022 we were unable to determine whether the alcohol industry had made any contributions to WHOF, because a third of WHOF’s published contributions were anonymised.^17^ When asked about this in a news interview in 2023, WHOF’s Chief Executive Officer responded that he was “committed to transparency” and that the foundation “will not accept a gift if there is a conflict of interest.”^120^ However, a recent analysis of funding disclosures made by WHOF from 2020 to 2023 found low and declining levels of transparency over time.^121^ This lack of transparency makes it extremely challenging to assess the degree of corporate influence on WHO or to hold WHOF accountable for its decisions, particularly as the organisations seek to increase private funding.

 Against a background of under-funding by member states, the UN’s partnerships with the alcohol industry place the system’s integrity and impartiality at risk. Ruckert and Labonté suggested that the ubiquity of PPPs in global health have led to a “deepening of the neoliberal management of individuals and populations, allowing private interest to become more embedded within the public sphere and to influence global and national health policy-making.”^122^ Considering the US government intends to withdraw from WHO,^123^ perceptions of the UN lacking independence could mean a further retreat of member states from the UN system. Diminishing contributions from member states would only then exacerbate the UN’s funding difficulties that PPPs were supposed to address, driving further reliance on private sector funding.

 To protect the UN’s independence and reputation, we strongly recommend that the UN system exclude the alcohol industry from all engagements, in line with the tobacco industry. The alcohol and tobacco industries have a long history of collaboration and co-ownership, using similar strategies to influence policy-making.^124^ The key difference between the two industries is that transnational tobacco corporations can no longer pursue UN partnerships or PPPs under WHO’s Framework Convention on Tobacco Control.^125^ Our findings here suggest the need for a similar international treaty on alcohol control, which would be an important safeguard against alcohol industry interference both globally and nationally. The UN also requires common standard procedures in due diligence when interacting with the private sector, which must be applied consistently across all organisations. These procedures should include clear steps to minimise personal and institutional conflicts of interest. Finally, public disclosure requirements are essential to ensure adequate transparency and accountability of all interactions between UN entities and the private sector.

## Conclusions

 Given alcohol remains a major threat to health, the UN’s widespread relationships with the alcohol industry undermine WHO’s mandate to promote health, as well as the system’s commitment to reduce the burden of NCDs as part of the 2030 Sustainable Development Agenda. These relationships are a key opportunity for the alcohol industry to build corporate citizenship and influence policy-making. While member states should equip the UN with the necessary resources, stronger safeguards are needed against alcohol industry interference and more effective mechanisms for transparency and accountability are essential. Excluding the alcohol industry from such partnerships would be an important first step.

## Disclosure of artificial intelligence (AI) use

 Not applicable.

## Ethical issues

 Ethics approval was not required as this work only involved analysis of publicly available material.

## Conflicts of interest

 Authors declare that they have no conflicts of interest.

## Supplementary files


Supplementary file 1 contains Tables S1-S3.
