# Supplementary material for: United Nations Partnerships With the Alcohol Industry
Source: Int J Health Policy Manag. 2026 Jan 28;15:8947. doi: 10.34172/ijhpm.8947 (PMC12980025; doi:10.34172/ijhpm.8947)
Supplement: Supplementary file 1 — contains Tables S1-S3. [file ijhpm-15-8947-s001.pdf]

Article title: United Nations Partnerships With the Alcohol Industry

Journal name: International Journal of Health Policy and Management (IJHPM)

Authors' information: June Yue Yan Leung*, Sally Casswell

SHORE and Whariki Research Centre, College of Health, Massey University, Auckland, New Zealand.

***Correspondence to:** June Yue Yan Leung; Email: y.leung@massey.ac.nz

Citation: Leung JYY, Casswell S. United Nations partnerships with the alcohol industry. Int J Health Policy Manag. 2025;14:8947. doi:10.34172/ijhpm.8947

Supplementary file 1

Table S1. United Nations (UN) entities included

Type of UN entity	Name of UN-affiliated entity	Website
Funds and programmes	UN Children's Fund (UNICEF)	https://www.unicef.org
	UN Development Programme (UNDP)	https://www.undp.org
	UN Environment Programme (UNEP)	https://www.unep.org
	UN Human Settlements Programme (UN-HABITAT)	https://unhabitat.org
	UN Populations Fund (UNFPA)	https://www.unfpa.org
	World Food Programme (WFP)	https://www.wfp.org
Specialised agencies	Food and Agriculture Organization (FAO)	https://www.fao.org
	International Bank for Reconstruction and Development (IBRD), WBG	https://www.worldbank.org/en/who-we-are/ibrd
	International Civil Aviation Organization (ICAO)	https://www.icao.int
	International Development Association (IDA), WBG	http://www.worldbank.org/ida
	International Finance Corporation (IFC), WBG	http://www.ifc.org
	International Fund for Agricultural Development (IFAD)	https://www.ifad.org
	International Labor Organization (ILO)	https://www.ilo.org
	International Maritime Organization (IMO)	https://www.imo.org
	International Monetary Fund (IMF)	https://www.imf.org
	International Telecommunication Union (ITU)	https://www.itu.int
	UN Educational, Scientific and Cultural Organization (UNESCO)	https://www.unesco.org
	UN Industrial Development Organization (UNIDO)	https://www.unido.org
	Universal Postal Union (UPU)	https://www.upu.int
	World Bank Group (WBG)	https://www.worldbank.org
	World Health Organization (WHO)	https://www.who.int
	World Intellectual Property Organization (WIPO)	https://www.wipo.int
	World Meteorological Organization (WMO)	https://public.wmo.int
	World Tourism Organization (UNWTO)	https://www.unwto.org
	Joint UN Programme on HIV/AIDS (UNAIDS)	http://www.unaids.org

Other entities and bodies	UN Entity for Gender Equality and the Empowerment of Women (UN Women)	http://www.unwomen.org
	UN High Commissioner for Refugees (UNHCR)	https://www.unhcr.org
	UN Institute for Disarmament Research (UNIDIR)	http://www.unidir.org
	UN Institute for Training and Research (UNITAR)	http://www.unitar.org
	UN Interregional Crime and Justice Research Institute (UNICRI)	https://unicri.it
	UN Office for Project Services (UNOPS)	https://www.unops.org
	UN Relief and Works Agency for Palestine Refugees in the Near East (UNRWA)	http://www.unrwa.org
	UN Research Institute for Social Development (UNRISD)	https://www.unrisd.org
	UN System Staff College (UNSSC)	https://www.unssc.org
	UN University (UNU)	https://unu.edu
Departments and offices	UN Department of Economic and Social Affairs (UNDESA) - Sustainable Development	https://sdgs.un.org/partnerships
	UN Office for Disaster Risk Reduction (UNDRR)	https://www.undrr.org
	UN Office for Partnerships (UNOP)	https://unpartnerships.un.org
	UN Office on Drugs and Crime (UNODC)	https://www.unodc.org
	UN Special Representative of the Secretary-General on Violence Against Children (SRSG/VAC)	https://violenceagainstchildren.un.org
Related organisations	Common Fund for Commodities (CFC)	https://www.common-fund.org
	International Organization for Migration (IOM)	http://www.iom.int
	International Trade Centre (ITC)	https://www.intracen.org
	Stop TB Partnership (hosted by UNOPS)	https://www.stoptb.org
	UN Capital Development Fund (UNCDF)	https://www.uncdf.org
	UN Framework Convention on Climate Change Secretariat (UNFCCC Secretariat)	https://unfccc.int
	UN Global Compact (UNGC)	https://unglobalcompact.org
	UN Interagency Task Force on NCDs	https://uniatf.who.int
	UN Office for the Coordination of Humanitarian Affairs (UNOCHA)	https://www.unocha.org
	UN System Chief Executives Board for Coordination (UNSCEB)	https://unsceb.org
	United Nations Conference on Trade and Development (UNCTAD)	https://unctad.org
	World Trade Organization (WTO)	https://www.wto.org
Related charitable organisations	Gavi, the Vaccine Alliance	https://www.gavi.org
	Joint SDG Fund	https://www.jointsdgfund.org
	The Global Fund	https://www.theglobalfund.org
	UN Foundation	https://unfoundation.org
	WHO Foundation	https://who.foundation

Supplementary Table S2. Transnational alcohol corporations (TNACs) included

Name of TNAC	Forbes Global 2000 rank[1]	Revenue (billions)[1]	Headquarters	Website
LVMH Moët Hennessy - Louis Vuitton (LVMH)	48	$93.10	France	https://www.lvmh.com
Anheuser-Busch InBev (AB InBev)	88	$59.40	Belgium	https://www.ab-inbev.com
Kweichow Moutai	269	$18.64	China	https://www.moutaichina.com
Diageo	314	$20.44	United Kingdom	https://www.diageo.com
Heineken	449	$32.83	Netherlands	https://www.heineken.com
Pernod Ricard	512	$12.55	France	https://www.pernod-ricard.com
Wuliangye Yibin	540	$10.32	China	https://www.wuliangye.com.cn
Asahi Group Holdings	648	$19.57	Japan	https://www.asahigroup-holdings.com
Constellation Brands	679	$9.96	United States	https://www.cbrands.com
Molson Coors Brewing	929	$12.00	United States	https://www.molsoncoors.com
Kirin Holdings	1000	$15.12	Japan	https://www.kirinholdings.com
Luzhou Lao Jiao	1056	$3.80	China	https://www.lzlj.com
Shanxi Xinghuacun Fen Wine Factory	1065	$4.00	China	https://www.fenjiu.com.cn
Jiangsu Yanghe Brewery	1242	$4.00	China	https://www.chinayanghe.com
Brown-Forman Corporation	1321	$4.20	United States	https://www.brown-forman.com
Carlsberg	1379	$10.68	Denmark	https://www.carlsberggroup.com
China Resources Beer Holdings	1580	$5.53	Hong Kong SAR, China	https://www.crbeer.com.hk
Thai Beverage (ThaiBev)	1627	$7.90	Thailand	https://www.thaibev.com

[1]Source: Murphy A, Schifrin M. Forbes 2024 Global 2000 List - The World's Largest Companies Ranked [Internet]. Forbes. 2024 [cited 2024 Sep 10]. Available from: https://www.forbes.com/lists/global2000/

Table S3. Event participation between United Nations (UN) entities and transnational alcohol corporations (TNACs)

UN entities involved	TNACs involved	Year	Country	Details	Relevant links
UNDP	Asahi	2023	Japan	UNDP in Tokyo hosted a roundtable meeting on business and human rights. Asahi was a participant.	https://www.undp.org/rolhr/news/bhr-ceo-roundtable-tokyo
UNDP, UNGC, UNOP	Diageo	2010	USA	A Diageo representative was a speaker at the UN summit on the Millennium Development Goals. Its corporate social responsibility initiatives were highlighted in the event's report.	https://unglobalcompact.org/news/75-10-26-2010<br><br>https://www.un.org/zh/mdg/summit2010/pdf/BusinessGuideMDGs.pdf
FAO, WHO	Diageo	2022	Italy	Senior representatives of Diageo India attended a meeting of the joint FAO/WHO Codex Alimentarius Commission Committee on Food Hygiene as part of the Indian delegation.	https://www.fao.org/fao-who-codexalimentarius/sh-proxy/en/?lnk=1&url=https%253A%252F%252Fworkspace.fao.org%252Fsites%252Fcodex%252FMeetings%252FCX-712-52%252FReport%252FREP22_FHe.pdf
FAO, WHO	Heineken	2021, 2022	Italy	A representative of Al ahram Beverage, a subsidiary of Heineken, attended meetings of the FAO/WHO Codex Alimentarius Commission Committee on Contaminants in Food as part of the Egyptian delegation.	https://www.fao.org/fao-who-codexalimentarius/sh-proxy/en/?lnk=1&url=https%253A%252F%252Fworkspace.fao.org%252Fsites%252Fcodex%252FMeetings%252FCX-735-15%252FREPORT%252FFINAL%252520REPORT%252FREP22_CF15e.pdf<br><br>https://www.fao.org/fao-who-codexalimentarius/sh-proxy/en/?lnk=1&url=https%253A%252F%252Fworkspace.fao.org%252Fsites%252Fcodex%252FMeetings%252FCX-701-44%252FFINAL%252520REPORT%252FRep21_CACe.pdf
FAO, WHO	Pernod Ricard	1997, 1998	Italy	A representative of Pernod Ricard participated in various meetings of the FAO/WHO Codex Alimentarium Commission, representing international business associations or as part of the French delegation.	https://www.fao.org/4/W5979E/w5979e0j.htm#list%20of%20participants%20%20%20liste%20des%20participants%20%20%20lista%20de%20participantes<br><br>https://www.fao.org/4/w5097e/w5097e0f.htm#bm15.1<br><br>https://www.fao.org/4/w9087e/w9087e0i.htm#bm18.1
FAO	LVMH	2021	Italy	A representative of Moët Hennessy was a speaker at the FAO's Global Symposium on Soil Biodiversity, at a session moderated by an FAO representative.	https://www.fao.org/fileadmin/user_upload/GSP/GSOBI21/Agenda_GSOBI21_Public.pdf
UNIDO	Luzhou Laojiao	2018	China	Luzhou Laojiao was invited to attend a summit on sustainability in Beijing hosted by the UNIDO Chinese Office, where they were awarded a "green enterprise award".	http://www.ce.cn/cysc/sp/jiu/201807/06/t20180706_29652028.shtml

WIPO	Wuliangye Yibin	2024	China	A representative of Wuliangye Yibin was a panelist at a forum moderated by WIPO in China on "United Nations 2030 Sustainable Development Goals: Role and Contribution of Geographical Indications and WIPO Lisbon System".	https://www.wipo.int/web/office-china/w/news/2024/wipo-china-geographical-indications-a-green-solution-for-sustainable-future
WIPO	AB InBev	2018	Belgium	A representative of AB InBev was a panelist at a seminar organised by WIPO.	https://www.wipo.int/edocs/mdocs/mdocs/en/wipo_rs_ip_bru_18/wipo_rs_ip_bru_18_inf_1_prov_3.pdf
WIPO	Pernod Ricard	2007	China	A representative of Pernod Ricard was a speaker at the International Symposium on Geographical Indications organised by WIPO and the Chinese State Administration for Industry and Commerce.	https://www.wipo.int/edocs/mdocs/geoind/en/wipo_geo_bei_07/wipo_geo_bei_07_inf_2.pdf
WIPO	LVMH, Pernod Ricard	2003	USA	Representatives of LVMH and Pernod Ricard were invited speakers at the Worldwide Symposium on Geographical Indications organised by WIPO and the US Patent and Trademark Office.	https://www.wipo.int/meetings/en/doc_details.jsp?doc_id=78732
WBG	AB InBev	2024	Virtual	A representative of AB InBev was a featured speaker on World Bank Live, World Bank's event streaming platform.	https://live.worldbank.org/en/experts/d/david-kamenetzky
WBG	AB InBev	2020	Virtual	A representative of AB InBev was a panelist at a webinar on digitisation of agribusiness in Africa organised by WBG.	https://www.worldbank.org/en/events/2020/11/18/digitization-of-agribusiness-payments-in-sub-saharan-africa-a-game-changer-for-farmers
UNAIDS	Heineken	2011	Netherlands	Heineken hosted a symposium on its work in Africa, with the UNAIDS Director of Partnerships in attendance.	https://www.unaids.org/en/resources/presscentre/featurestories/2011/april/20110406heineken
UNAIDS	Heineken	2007	Netherlands	Heineken hosted a symposium on “the future of health care in resource-poor settings” that invited the UNAIDS Executive Director as a guest speaker.	https://www.unaids.org/en/resources/presscentre/featurestories/2007/october/20071003businessandaids
UN Women	AB InBev (SABMiller)	2014	Switzerland	UN Women’s Executive Director attended a reception hosted by SABMiller at the World Economic Forum in Davos.	https://www.unwomen.org/en/news/stories/2014/1/ed-to-attend-wef-annual-meeting-in-davos
UN Women, UNGC	Carlsberg	2012	Serbia	Carlsberg was one of the private sector champions identified by UN Women. They also attended an event on women's empowerment organised by UN Women and UNGC.	https://www.unwomen.org/en/news/stories/2012/4/12-ceos-sign-the-women-s-empowerment-principles-in-the-presence-of-serbia-s-president

UNITAR	AB InBev	2023	Virtual	UNITAR hosted an online event in support of the UN Road Safety Week and invited AB InBev to speak.	https://www.unitar.org/about/news-stories/news/unitar-and-its-cifal-network-host-global-online-dialogue-support-un-road-safety-week
UNITAR	AB InBev, Diageo, Heineken, Pernod Ricard	2023	USA	UNITAR hosted a side-event at the UN 2023 Water Conference and invited speakers from AB InBev, Diageo, Heineken and Pernod Ricard.	https://www.unitar.org/about/news-stories/news/unitar-participates-un-2023-water-conference
UNITAR	AB InBev	2021	Virtual	UNITAR hosted a master class on road safety for senior government officials around the world. AB InBev was one of the presenters.	https://www.unitar.org/about/news-stories/news/connecting-asia-pacific-smart-safe-and-resilient-roads
UNITAR	AB InBev	2019	Switzerland	UNITAR hosted a roundtable meeting on "successful partnerships in support of the SDGs" at the World Economic Forum Annual Meeting in Davos. This event highlighted the partnership between UNITAR and AB InBev.	https://www.unitar.org/about/news-stories/news/unitar-hosted-dialogue-successful-partnerships-support-sdgs
UNITAR	AB InBev	2018	India	UNITAR hosted a road safety conference in Gurugram, with a representative of AB InBev as a panelist. AB InBev also sponsored a road safety data dashboard for Gurugram and is a member of the Safer Roads for Gurugram Initiative Working Group hosted by UNITAR.	https://www.unitar.org/about/news-stories/news/unitar-hosts-road-safety-conference-support-safer-roads-gurugram
UNDESA, UN Water	Wuliangye Yibin	2023	USA	A Wuliangye representative presented at the UNSDG Summit in 2023, organised by UNDESA and UN Water, as one of the best case studies of biodiversity.	https://new.qq.com/rain/a/20230920A00RPQ00
UNDESA	Pernod Ricard	2017	USA	Paul Ricard Oceanography Institute, owned by Pernod Ricard, attended the first UN Ocean Conference as part of the French delegation.	https://assets.pernod-ricard.com/2017_06_09_iopr_at_the_un.pdf
UNDRR	Heineken	2023	USA	A representative of Heineken was a panelist at a UN Water Conference side-event organised by UNDRR.	https://www.undrr.org/event/infrastructure-resilience-prerequisite-ensuring-availability-and-sustainable-water-management
UNFCCC	Kirin	1997	Japan	Kirin was one of two companies representing Japan at the third session of the Conference of the Parties to the UN Framework Convention on Climate Change in Kyoto.	https://www.kirinholdings.com/en/investors/files/pdf/environmental2023e.pdf
UNFCCC, UNIDO	AB InBev	2018	Uruguay	A representative of AB InBev was invited to present at a regional technical expert meeting on circular economy and waste-to-energy organised by UNFCCC in collaboration with UNIDO.	https://unfccc.int/ttclear/events/2018_event7

UNCTAD	Pernod Ricard	2018	Belgium	UNCTAD hosted a seminar on EU free trade agreements, at which a Pernod Ricard representative was a speaker.	https://unctad.org/meeting/seminar-use-eus-free-trade-agreements
UNCTAD, ITC	AB InBev (SABMiller)	2014	Switzerland	A representative of SAB Miller was a speaker at the UNCTAD's World Investment Forum, moderated by ITC.	https://intracen.org/news-and-events/news/empowering-the-poor-to-benefit-from-global-value-chains
UNOCHA	Diageo	2015	UK	Diageo was a facilitator at a business consultation meeting hosted by UNOCHA and Business in the Community.	https://www.unocha.org/publications/report/world/business-community-s-feedback-report-un-ocha-business-collaboration-humanitarian-action
WTO	AB InBev, SAB Miller, Carlsberg, Heineken	2015	Switzerland	A side-event was held at the WTO's 5th Global Review of Aid for Trade conference by the Worldwide Brewing Alliance for informal exchange between country delegates and four major alcohol corporations (SABMiller, AB InBev, Carlsberg and Heineken).	https://www.wto.org/english/tratop_e/devel_e/a4t_e/global_review15prog_e/5gr_review_e.pdf
WTO	AB InBev, SABMiller, Carlsberg, Heineken	2013	Switzerland	A side-event was held at the WTO's 4th Global Review of Aid for Trade conference by the Worldwide Brewing Alliance for informal exchange between country delegates and four major alcohol corporations (SABMiller, AB InBev, Carlsberg and Heineken).	https://www.wto.org/english/tratop_e/devel_e/a4t_e/4th_aft_summary_report_side_events_e_v4.pdf
WTO	Diageo	2020	Switzerland	WTO hosted a business leaders meeting to discuss the role of trade policy in COVID-19. Diageo was represented at this event.	https://www.wto.org/english/news_e/news20_e/trdia_19may20_e.htm
WTO	Diageo	2018	Switzerland	WTO hosted a business leaders meeting to discuss how the WTO can help in dealing with challenges in conducting trade operations. Diageo was represented at this event.	https://www.wto.org/english/news_e/news18_e/trdia_07jun18_e.htm
WTO	Diageo	2016	Switzerland	WTO hosted a business leaders meeting to discuss how the WTO can help in dealing with challenges in conducting trade operations. Diageo was represented at this event.	https://www.wto.org/english/news_e/news16_e/bus_30may16_e.htm
WTO	Pernod Ricard	2021	Virtual	WTO hosted a virtual trade dialogue with business leaders to discuss issues to be taken up at the WTO's 12th Ministerial Conference. Pernod Ricard was represented.	https://www.wto.org/english/news_e/news21_e/trdia_23jun21_e.htm
WTO	Pernod Ricard	2019	Switzerland	A representative of Pernod Ricard was a speaker at the WTO Public Forum.	https://www.wto.org/english/forums_e/public_forum19_e/draft_programme_e.pdf

WTO	Pernod Ricard	2019	Switzerland	A representative of Pernod Ricard was a panelist at the Aid for Trade Global Review hosted by WTO.	https://www.wto.org/english/tratop_e/devel_e/a4t_e/gr19_e/glossy_summary_report_e.pdf
WTO	Pernod Ricard	2017	Switzerland	A representative of Pernod Ricard was a speaker at a WTO conference on e-commerce.	https://www.wto.org/english/res_e/reser_e/datadigitaleco17_e.htm
WTO	Pernod Ricard	2012	Switzerland	A representative of Pernod Ricard was a speaker at the WTO Public Forum.	https://www.wto.org/english/forums_e/public_forum12_e/programme_e.pdf
Stop TB Partnership	AB InBev	2013	Switzerland	A senior executive of ABInBev, also chair of the UN Innovation Working Group on Women and Children's Health, was a panelist at a global health forum hosted by Stop TB Partnership.	https://www.stoptb.org/event/post-2015-consultation-global-health-and-diplomacy
Global Fund	Heineken	2008 – 2019	Various	As early as 2008, Heineken has participated in the Global Fund's annual board meetings. Heineken still participated in the board meeting in 2019, even after its partnership with the Global Fund was suspended.	https://archive.theglobalfund.org/media/3717/archive_bm18-19participants_list_en.pdf https://archive.theglobalfund.org/media/8543/archive_bm41-participant_list_en.pdf
UN General Assembly	AB InBev	2023	USA	AB InBev participated in the 78th UN General Assembly.	https://www.ab-inbev.com/news-media/news-stories/ab-inbev-taps-into-the-power-of-beer-and-partnerships-to-grow-economies-advance-the-un-sustainable-development-goals
UN Office in Geneva	Luzhou Laojiao	2019	Switzerland	Luzhou Laojiao held an exhibition/tasting session at the Palais des Nations in Geneva as part of an event promoting Sichuan culture co-hosted by the UN Office at Geneva and the Sichuan provincial government.	https://www.arp-creative.com/zh/work/luzhou-laojiao-tasting-banquet/
UN Resident Coordinator Office in China	Wuliangye Yibin	2023	China	A representative of Wuliangye was a speaker at the 5th Belt and Road Women's Forum hosted by the UN Resident Coordinator Office in China and several other organisations.	https://www.wuliangye.com.cn/zh/main/main.html#/g=NEWS&id=34&dId=11763
